# Effects of Iloprost on TRPM7-Mediated Apoptosis and Oxidative Stress in an Experimental Testicular Torsion–Detorsion Model

**DOI:** 10.3390/ijms27167454

**Published:** 2026-08-20

**Authors:** Nalan Kaya Tektemur, Aysenur Bilgetay Unlu, Merve Kavak Balgetir, Ahmet Tektemur, Gonca Ozan Kocamuftuoglu, Ramazan Fazil Akkoc, Osman Fatih Yilmaz, Esra Aydemir, Tuncay Kuloglu

**Affiliations:** 1 Department of Histology and Embryology, Faculty of Medicine, Firat University, Elaziğ 23119, Türkiye; abilgetay47@gmail.com (A.B.U.); esradvc23@hotmail.com (E.A.); tkuloglu@firat.edu.tr (T.K.); 2 Department of Histology and Embryology, Elazig Fethi Sekin City Hospital, Elaziğ 23280, Türkiye; mkavakbalgetir23@gmail.com; 3 Department of Medical Biology, Faculty of Medicine, Firat University, Elaziğ 23119, Türkiye; atektemur@firat.edu.tr; 4 Department of Biochemistry, Faculty of Medicine, Burdur Mehmet Akif Ersoy University, Burdur 15030, Türkiye; gokocamuftuoglu@mehmetakif.edu.tr; 5 Department of Anatomy, Faculty of Medicine, Kutahya Health Sciences University, Kütahya 43020, Türkiye; ramazan_fazil@hotmail.com; 6 Pathology Laboratory Techniques, Faculty of Medicine, Mus Alparslan University, Muş 49250, Türkiye; of.yilmaz@alparslan.edu.tr

**Keywords:** testicular torsion, ischemia–reperfusion injury, TRPM7, iloprost, oxidative stress, germ cell apoptosis

## Abstract

Testicular torsion is a urological emergency that causes ischemia–reperfusion (I/R) injury and may result in irreversible germ cell loss and impaired fertility. Oxidative stress, inflammation, and apoptotic pathways play central roles in its pathogenesis. Transient Receptor Potential Melastatin-7 (TRPM7), a bifunctional ion channel/kinase, has recently been implicated in I/R-related cellular injury. Iloprost, a prostacyclin analogue with vasodilatory and antioxidant properties, may offer protective effects; however, its impact on TRPM7-mediated pathways in testicular I/R injury remains unclear. This study aimed to investigate the effects of iloprost on oxidative stress, apoptosis, and TRPM7 expression in an experimental testicular torsion–detorsion model. Thirty-five male Sprague Dawley rats were randomized into five groups: control, sham, iloprost, torsion–detorsion (T/D), and T/D + iloprost. After 60 min of torsion and subsequent detorsion, iloprost (2 µg/kg, intraperitoneal) was administered in the treatment group. Testicular tissues and serum samples were analyzed after 48 h. Histopathology (H&E), apoptosis (TUNEL assay), TRPM7 immunohistochemistry and mRNA expression (RT-qPCR), and serum total antioxidant status (TAS) and total oxidant status (TOS) were evaluated. Torsion–detorsion significantly increased TOS levels, apoptotic cell ratio (ACR), TRPM7 expression, and Bax mRNA expression, while reducing TAS levels (*p* < 0.05). Iloprost administration significantly improved oxidative stress parameters, restoring TAS and reducing TOS compared with the untreated T/D group. However, it did not significantly reduce histopathological damage, ACR, TRPM7 expression, or Bax mRNA expression. Bcl-2 expression remained largely unchanged across groups. Iloprost improved systemic oxidative status in testicular I/R injury but did not significantly attenuate TRPM7 expression, apoptotic changes, or structural damage under the experimental conditions used. The concurrent increase in TRPM7 expression and apoptotic indices following torsion–detorsion suggests an association between these alterations; however, the present study does not establish a causal relationship between TRPM7 and apoptosis. As TRPM7 activity was not directly assessed or experimentally manipulated, further studies involving TRPM7 inhibition or genetic silencing are required to determine its mechanistic role in testicular I/R-induced apoptosis.

## 1. Introduction

Testicular torsion is a serious urological emergency that can lead to ischemia and subsequent necrosis of the testis, necessitating prompt surgical intervention. The pathological process initially involves obstruction of venous outflow, which is followed by compromised arterial perfusion, ultimately resulting in testicular infarction [1,2]. Both experimental and clinical evidence indicate that spermatogenic and Sertoli cells sustain irreversible damage within approximately six hours of torsion, whereas Leydig cells exhibit relatively greater resistance to ischemia and are typically affected after about ten hours [3,4].

Timely detorsion remains the cornerstone of management, as it restores blood flow and enhances the likelihood of testicular salvage. Nevertheless, the sequence of ischemia followed by reperfusion gives rise to testicular ischemia–reperfusion (I/R) injury, which represents the central pathophysiological mechanism underlying testicular torsion [5,6]. Although reperfusion is essential for tissue survival, it may paradoxically exacerbate cellular injury—a phenomenon referred to as reperfusion injury [7,8].

Reperfusion of the ipsilateral testis is characterized by leukocyte infiltration and the excessive generation of reactive oxygen species (ROS), along with increased production of pro-inflammatory cytokines and adhesion molecules [9,10,11]. These events promote lipid peroxidation, DNA damage, and disruption of cellular integrity, ultimately impairing testicular function. Apoptosis constitutes a key component of testicular I/R injury. The heightened oxidative stress and inflammatory milieu activate the mitochondrial apoptotic pathway, where an increased Bax/Bcl-2 ratio and activation of caspase-3 are closely associated with germ cell loss and impaired spermatogenesis [12,13,14]. Furthermore, disruption of the blood–testis barrier and the potential development of autoimmune responses may also contribute to damage in the contralateral testis, underscoring the broader implications of this condition on male fertility.

Transient Receptor Potential Melastatin-7 (TRPM7) is a unique bifunctional protein that comprises both an ion channel and a serine/threonine protein kinase domain, enabling its participation in diverse cellular processes such as growth, differentiation, migration, and survival [15,16]. TRPM7 is widely expressed in endothelial cells, monocytes, neurons, osteoblasts, mesenchymal stem cells, and vascular smooth muscle cells [17,18]. As a kinase, it phosphorylates substrates including annexin I, myosin II, and m-calpain, while as an ion channel, it conducts essential divalent cations such as Ca^2+^, Mg^2+^, and Zn^2+^ [15,19].

Cells deficient in TRPM7 exhibit intracellular Mg^2+^ depletion and reduced viability, which can be rescued by extracellular Mg^2+^ supplementation [20,21]. In addition to its role in cellular homeostasis, TRPM7 has been increasingly implicated in inflammatory and ischemic processes, influencing the proliferation of lymphocytes, endothelial cells, and cancer cells. Under conditions of metabolic stress such as hypoxia or oxygen–glucose deprivation, TRPM7-associated currents may promote excessive divalent cation influx and contribute to cellular injury. Experimental suppression of TRPM7 has been shown to reduce anoxic Ca^2+^ uptake, ROS production, and neuronal cell death, providing functional evidence for the involvement of TRPM7 in ischemia-associated cell injury [15]. Importantly, evidence from renal ischemia–reperfusion models further supports a relationship between TRPM7 and apoptotic signaling. Increased TRPM7 expression and TRPM7-like currents have been reported following hypoxia/reoxygenation and renal I/R injury, whereas TRPM7 silencing reduced caspase-3 activation, the BAX/BCL-2 ratio, apoptosis, and tissue injury [16]. These findings suggest that TRPM7 may contribute to apoptotic cell injury under ischemic conditions; however, its functional role in apoptosis following testicular torsion–detorsion remains unclear.

Although several calcium-permeable TRP channels have been implicated in ischemia–reperfusion injury, TRPM7 was specifically selected as the principal molecular target in the present study because of its distinctive dual function as a divalent cation-permeable channel and serine/threonine kinase, its critical role in Ca^2+^ and Mg^2+^ homeostasis, and its previously reported involvement in cellular responses to hypoxia, oxidative stress, and ischemic injury. Furthermore, experimental evidence demonstrating attenuation of apoptotic injury following TRPM7 suppression or silencing provides an additional rationale for investigating whether alterations in TRPM7 expression accompany apoptotic changes following testicular torsion–detorsion [15,17].

Iloprost, a synthetic and stable analogue of prostacyclin, has potent vasodilatory and anti-aggregatory properties and exerts additional endothelial-protective, anti-inflammatory, and cytoprotective effects relevant to ischemia–reperfusion injury [18,19]. Experimental evidence also indicates that iloprost may influence apoptotic signaling during I/R injury; in a renal I/R model, iloprost reduced caspase-3, caspase-8, and caspase-9 activities, suggesting modulation of both death receptor-associated and mitochondrial apoptotic pathways [20]. However, there is currently no evidence demonstrating that iloprost directly targets, inhibits, or otherwise modulates TRPM7 channel activity. Therefore, iloprost was not used in the present study as a TRPM7 inhibitor. Rather, it was selected as a pharmacological intervention because its established effects on oxidative stress, inflammation, endothelial function, and microcirculation may modify the cellular environment associated with reperfusion injury.

Accordingly, we hypothesized that testicular torsion–detorsion would be accompanied by alterations in TRPM7 expression and apoptotic indices and that the protective effects of iloprost on the I/R environment might be accompanied by changes in these parameters. Therefore, the present study aimed to evaluate oxidative status, TRPM7 expression, and apoptosis following experimental testicular torsion–detorsion and to determine how these parameters are affected by iloprost treatment. Importantly, the study was designed to assess TRPM7 expression and its association with apoptotic changes rather than to establish a direct TRPM7-dependent apoptotic mechanism.

## 2. Results

### 2.1. Serum TAS and TOS Levels

**Figure 1:**
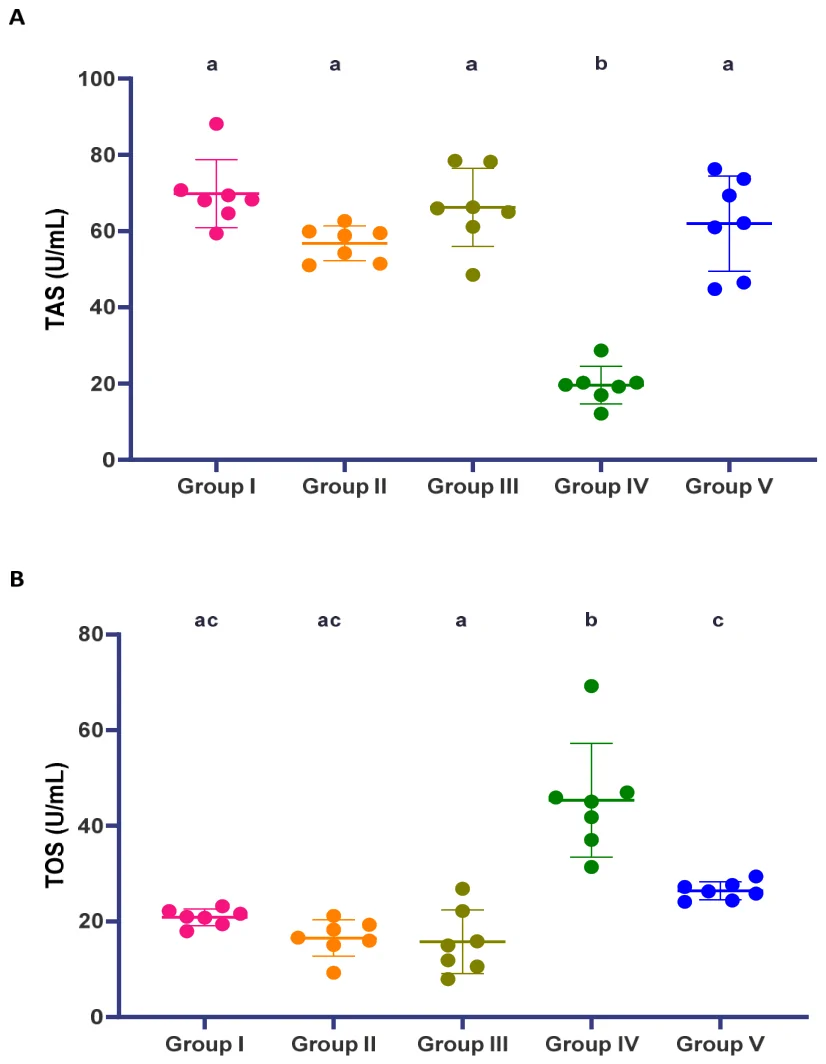
**Serum total antioxidant status (TAS) and total oxidant status (TOS) levels in the experimental groups:** (**A**) Serum TAS levels and (**B**) serum TOS levels. Individual points represent individual animals (*n* = 7 per group), and data are presented as mean ± SD. Statistical comparisons were performed using one-way ANOVA followed by Tukey’s multiple-comparison test. Different lowercase letters indicate statistically significant differences between groups (*p* < 0.05), whereas groups sharing at least one letter are not significantly different.

Serum TAS and TOS levels differed significantly among the experimental groups (TAS: one-way ANOVA, F(4,30) = 37.45, *p* < 0.0001; TOS: F(4,30) = 24.96, *p* < 0.0001). Tukey’s multiple-comparison test showed that TAS levels were significantly lower in Group IV than in Groups I, II, III, and V (all adjusted *p* < 0.0001), whereas no significant differences were observed among Groups I, II, III, and V (all adjusted *p* > 0.05) (Figure 1A). Conversely, TOS levels were significantly higher in Group IV than in all other groups (all adjusted *p* < 0.0001).

TOS levels in Group V were also significantly higher than those in Group III (adjusted *p* = 0.0314), while Group V did not differ significantly from Groups I or II (adjusted *p* = 0.5021 and *p* = 0.0530, respectively) (Figure 1B).

### 2.2. Histopathological Findings

**Figure 2:**
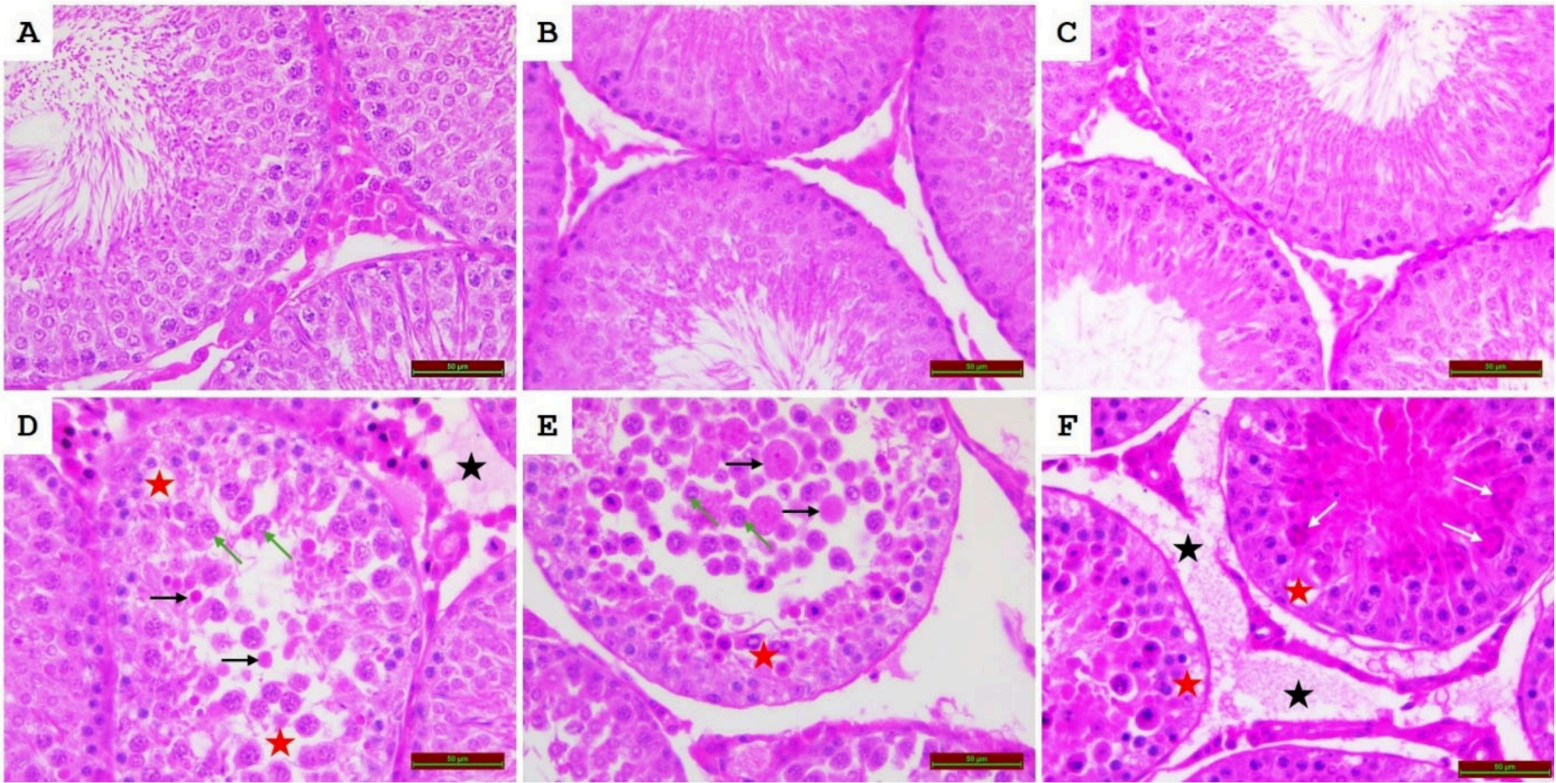
**Histopathological evaluation of testicular tissues in experimental groups (H&E staining, ×40).** Normal histological architecture was observed in Groups I (**A**), II (**B**), and III (**C**). In Group IV (**D**), testicular sections demonstrated prominent edema (black asterisk), degeneration of the germinal epithelium (red asterisk), atypical residual bodies (black arrow), and germ cell desquamation (green arrow). In Group V (**E**,**F**), findings similar to those observed in Group IV, including edema (black asterisk), degeneration of the germinal epithelium (red asterisk), atypical residual bodies (black arrow), germ cell desquamation (green arrow), and multinucleated giant cells (white arrow), were detected. Scale bar = 50 µm.

**Figure 3:**
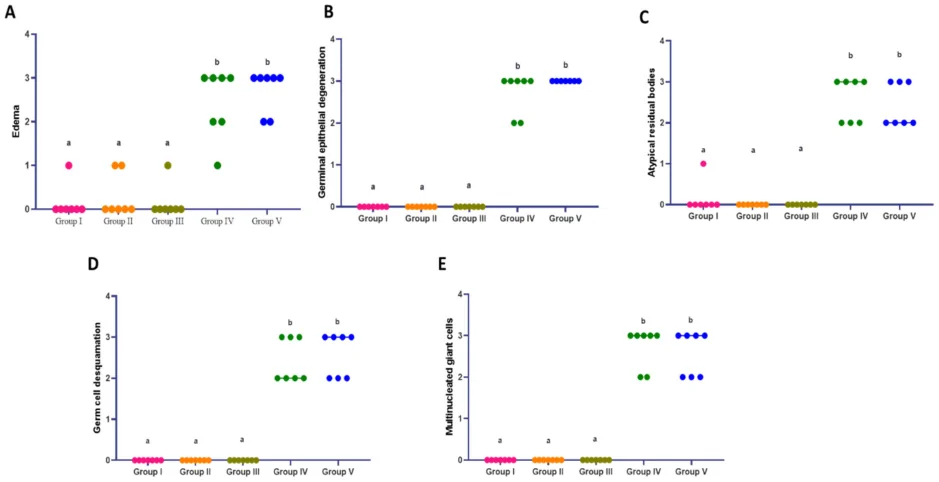
**Individual histopathological scores of testicular tissue among the experimental groups:** (**A**) Edema, (**B**) germinal epithelial degeneration, (**C**) atypical residual bodies, (**D**) germ cell desquamation, and (**E**) multinucleated giant cells. Individual data points represent individual animals (*n* = 7 per group), and horizontal lines indicate the median. Histopathological alterations were scored semi-quantitatively on a 0–3 scale. Different lowercase letters indicate statistically significant differences between groups according to Dunn’s multiple-comparison test following the Kruskal–Wallis test (*p* < 0.05); groups sharing the same letter are not significantly different.

Histological examination of hematoxylin and eosin (H&E)-stained sections under light microscopy revealed several pathological alterations, including interstitial edema, degeneration of the germinal epithelium, the presence of atypical residual bodies, sloughing of germ cells into the tubular lumen, and multinucleated giant cells. These histopathological changes were minimal and comparable in Group I, Group II, and Group III. In contrast, the severity of these alterations was markedly increased in Group IV and Group V (Figure 2). The distribution of individual histopathological scores of testicular tissue across the experimental groups is presented in Figure 3.

### 2.3. TRPM7 Immunoreactivity

**Figure 4:**
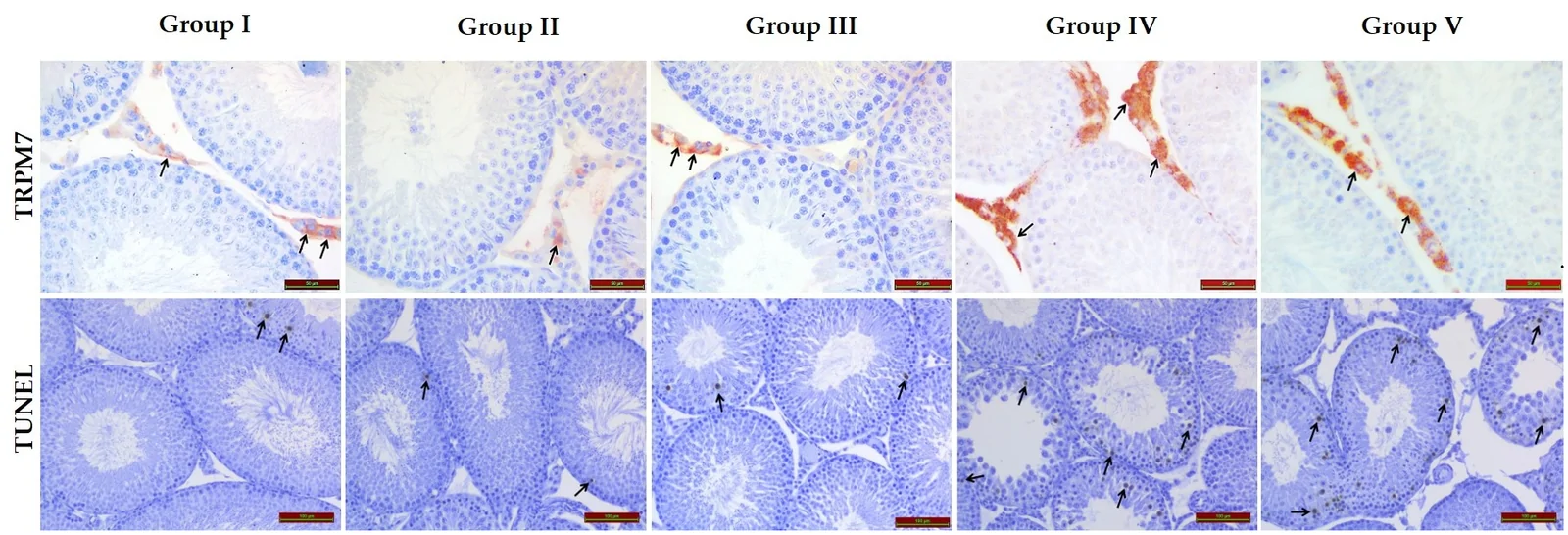
Testicular TRPM7 immunoreactivity and TUNEL staining in the experimental groups. TRPM7 immunoreactivity was evaluated at ×40 magnification and TUNEL staining at ×20 magnification. Data are presented as medians (min–max). Black arrows indicate cytoplasmic positive immunostaining in TRPM7 immunohistochemistry, while in TUNEL staining they indicate apoptotic cells with brown-stained nuclei. Scale bar = 50 µm.

**Figure 5:**
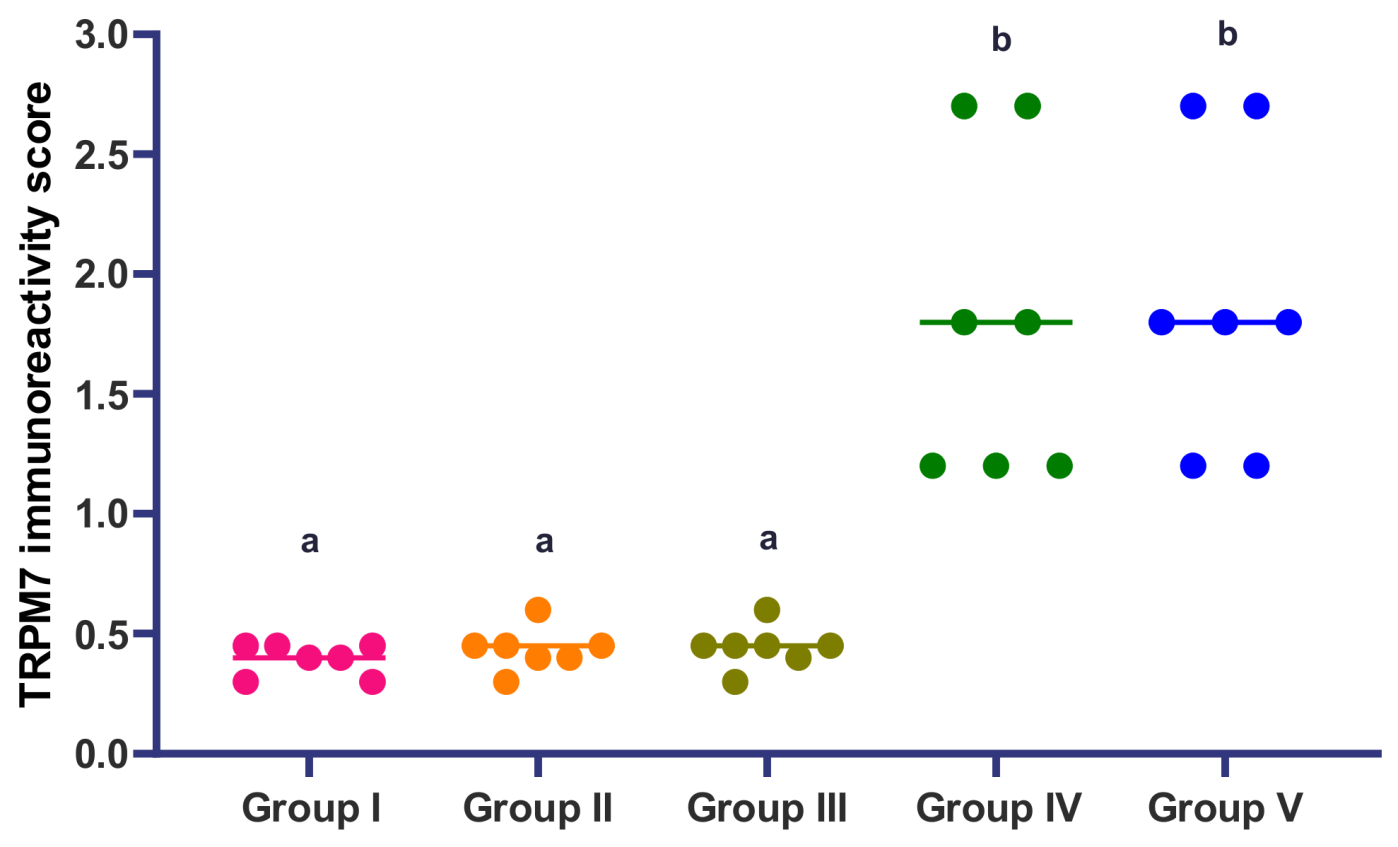
**Testicular TRPM7 immunoreactivity score in the experimental groups.** Individual data points represent individual animals (*n* = 7 per group), and horizontal lines indicate the median. Different lowercase letters indicate statistically significant differences between groups according to Dunn’s multiple-comparison test following the Kruskal–Wallis test (*p* < 0.05); groups sharing the same letter are not significantly different.

Immunohistochemical analysis demonstrated that TRPM7 immunoreactivity was predominantly localized in the interstitial areas of the testicular tissue (Figure 4). TRPM7 immunoreactivity differed significantly among the experimental groups (Kruskal–Wallis test, H = 25.88, *p* < 0.0001). Dunn’s multiple-comparison test showed significantly higher TRPM7 immunoreactivity in Groups IV and V than in Groups I, II, and III (all adjusted *p* < 0.05). No significant difference was observed between Groups IV and V or among Groups I, II, and III (all adjusted *p* > 0.05) (Figure 5).

### 2.4. TUNEL Assay Findings

**Figure 6:**
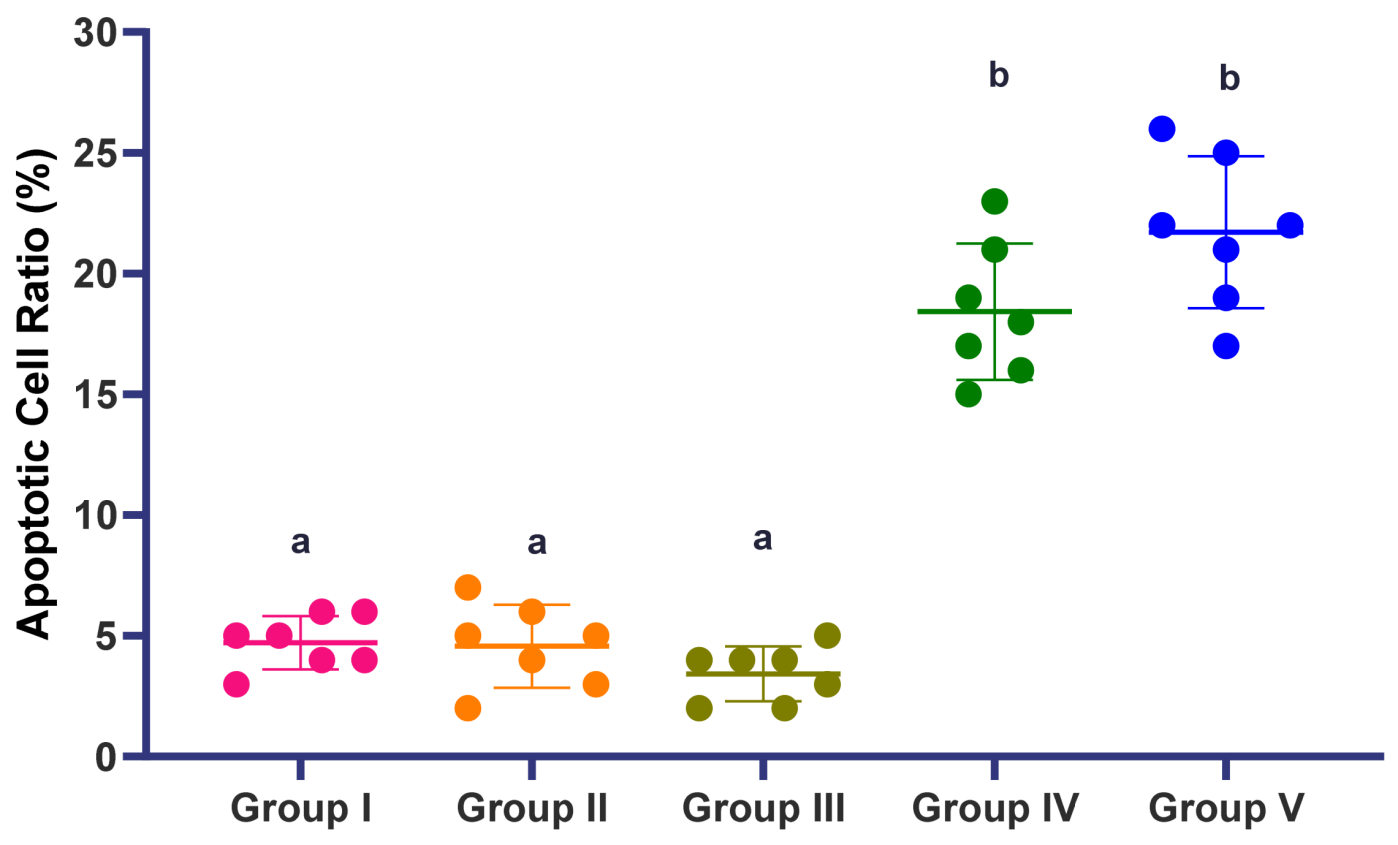
**Apoptotic index (ACR) in the experimental groups.** Individual data points represent individual animals (*n* = 7 per group), and horizontal lines with error bars indicate the mean ± SD. Different lowercase letters indicate statistically significant differences between groups according to Tukey’s multiple-comparison test following one-way ANOVA (*p* < 0.05); groups sharing the same letter are not significantly different.

TUNEL staining was performed to assess apoptosis in testicular tissue. TUNEL-positive cells were predominantly observed in the seminiferous tubular epithelium (Figure 4). The apoptotic cell ratio (ACR) differed significantly among the experimental groups (one-way ANOVA, F(4,30) = 115.2, *p* < 0.0001). Tukey’s multiple-comparison test showed that the ACR was significantly higher in Groups IV and V than in Groups I, II, and III (all adjusted *p* < 0.0001). No significant difference was observed between Groups IV and V (adjusted *p* = 0.0566), and no significant differences were detected among Groups I, II, and III (all adjusted *p* > 0.05). The ACR values for all groups are summarized in Figure 6.

### 2.5. Quantitative RT-PCR Results

**Figure 7:**
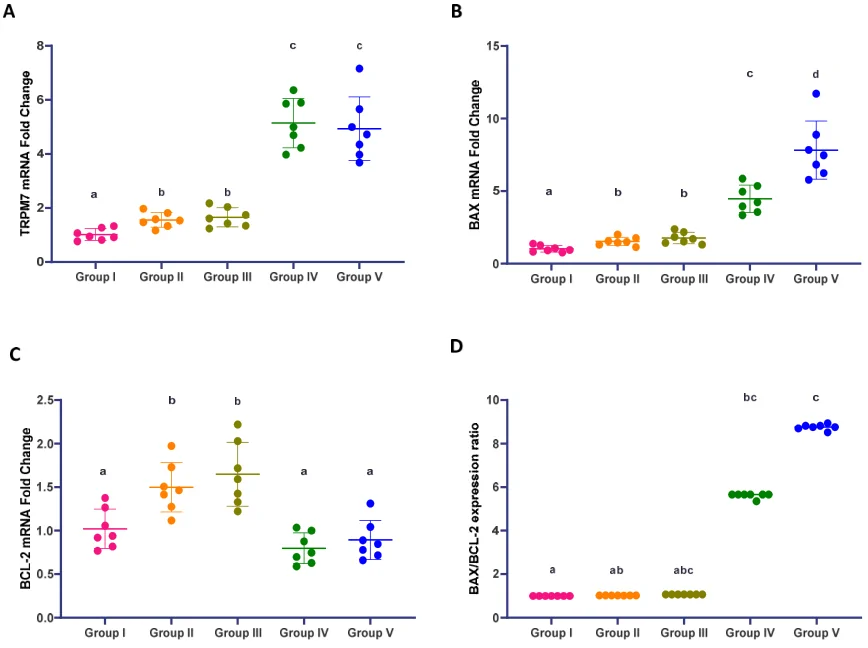
**RT-qPCR analysis of TRPM7, BAX, and BCL-2 mRNA expression and the relative BAX/BCL-2 expression ratio in the experimental groups:** (**A**) TRPM7 mRNA relative expression, (**B**) BAX mRNA relative expression, (**C**) BCL-2 mRNA relative expression, and (**D**) relative BAX/BCL-2 expression ratio. Individual points represent individual animals (*n* = 7 per group). Relative mRNA expression was calculated using the 2^−ΔΔCt^ method, with Group I serving as the calibrator. For TRPM7, BAX, and BCL-2, statistical comparisons were performed on ΔCt values using one-way ANOVA followed by Tukey’s multiple-comparison test; relative expression values are graphically presented as individual data points with mean ± SD. The BAX/BCL-2 expression ratio was calculated for each animal from the corresponding relative expression values. Statistical comparisons of the BAX/BCL-2 ratio were performed on log_2_-transformed ratio values using the Kruskal–Wallis test followed by Dunn’s multiple-comparison test; individual ratio values are graphically presented with the median. Different lowercase letters indicate statistically significant differences between groups (*p* < 0.05); groups sharing at least one letter are not significantly different.

qRT-PCR analysis revealed significant differences in TRPM7, BAX, and BCL-2 mRNA expression among the experimental groups. TRPM7 mRNA expression differed significantly among groups (one-way ANOVA, F(4,30) = 93.24, *p* < 0.0001). Tukey’s multiple-comparison test performed on ΔCt values showed that TRPM7 expression was significantly increased in Groups IV and V compared with Groups I, II, and III (all adjusted *p* < 0.0001). Groups II and III also showed significantly higher TRPM7 expression than Group I (adjusted *p* = 0.0032 and *p* = 0.0008, respectively). No significant differences were observed between Groups II and III (adjusted *p* = 0.9850) or between Groups IV and V (adjusted *p* = 0.9895) (Figure 7A).

BAX mRNA expression also differed significantly among groups (one-way ANOVA, F(4,30) = 105.7, *p* < 0.0001). BAX expression was significantly increased in Groups IV and V compared with Groups I, II, and III (all adjusted *p* < 0.0001). Moreover, BAX expression was significantly higher in Group V than in Group IV (adjusted *p* = 0.0003). Groups II and III also showed significantly higher BAX expression than Group I (adjusted *p* = 0.0102 and *p* = 0.0004, respectively), whereas no significant difference was observed between Groups II and III (adjusted *p* = 0.7470) (Figure 7B).

BCL-2 mRNA expression differed significantly among groups (one-way ANOVA, F(4,30) = 16.02, *p* < 0.0001). Tukey’s multiple-comparison test showed significantly higher BCL-2 expression in Groups II and III than in Group I (adjusted *p* = 0.0157 and *p* = 0.0020, respectively). BCL-2 expression was also significantly higher in Group II than in Groups IV and V (adjusted *p* < 0.0001 and *p* = 0.0006, respectively) and in Group III than in Groups IV and V (both adjusted *p* < 0.0001). No significant differences were observed between Groups I and IV (adjusted *p* = 0.1929), Groups I and V (adjusted *p* = 0.7452), Groups II and III (adjusted *p* = 0.9324), or Groups IV and V (adjusted *p* = 0.8399) (Figure 7C).

The relative BAX/BCL-2 expression ratio differed significantly among the experimental groups (Kruskal–Wallis test, H = 33.46, *p* < 0.0001). Dunn’s multiple-comparison test showed that the BAX/BCL-2 ratio was significantly higher in Group IV than in Group I (adjusted *p* = 0.0010) and in Group V than in Groups I (adjusted *p* < 0.0001) and II (adjusted *p* = 0.0010). No significant difference was observed between Groups IV and V (adjusted *p* > 0.9999). The remaining pairwise comparisons were also not statistically significant (Figure 7D).

## 3. Discussion

In the present study, serum total antioxidant status (TAS) levels were significantly decreased, whereas total oxidant status (TOS) levels were significantly increased in the testicular torsion–detorsion (T/D) group. Administration of iloprost restored TAS and TOS levels to values comparable with those of the control group. However, iloprost treatment did not result in significant improvements in histopathological alterations, TRPM7 immunoreactivity, or apoptosis induced by testicular torsion.

The experimental model of testicular torsion–detorsion represents a well-established paradigm of ischemia–reperfusion (I/R) injury, in which germ cells are among the most susceptible cell populations. Tissue hypoxia followed by reperfusion leads to oxidative stress, triggering irreversible apoptotic processes in germ cells. Numerous studies have demonstrated that reactive oxygen species (ROS) are major contributors to tissue damage induced by I/R injury. Furthermore, excessive ROS production not only induces lipid peroxidation and DNA damage but also disrupts mitochondrial integrity, thereby amplifying apoptotic signaling pathways [9,21].

Beyond its role in maintaining ionic homeostasis, TRPM7 has been proposed to function as a cellular stress sensor linking oxidative stress with intracellular ion regulation and cell survival. Previous studies suggest that, under ischemic or hypoxic conditions, oxidative stress may influence TRPM7 channel activity and contribute to disturbances in intracellular Ca^2+^ and Mg^2+^ homeostasis. Such ionic disturbances have been associated with mitochondrial dysfunction, impaired ATP production, enhanced ROS generation, and activation of apoptotic signaling pathways. Importantly, functional studies provide evidence that TRPM7 may contribute to cell death rather than merely accompany it. Aarts et al. demonstrated that suppression of TRPM7 expression reduced TRPM7-associated currents, anoxic Ca^2+^ uptake, ROS production, and neuronal death [16]. Similarly, in renal I/R-related injury, TRPM7 silencing reduced caspase-3 activation, the BAX/BCL-2 ratio, apoptosis, and tissue injury [17]. These findings provide experimental support for a functional relationship between TRPM7 activity and apoptotic injury in other ischemic/hypoxic models.

In the present study, however, TRPM7 was assessed only at the mRNA and immunohistochemical expression levels, and neither TRPM7 channel activity nor TRPM7 silencing/inhibition was investigated. Therefore, although our findings can be interpreted within this biologically plausible framework, they do not demonstrate that TRPM7 is mechanistically upstream of apoptosis in testicular torsion–detorsion injury. Direct inhibition or genetic silencing of TRPM7 would be required to establish such a causal relationship in testicular tissue.

Previous investigations of testicular I/R injury have reported structural disruption of the seminiferous tubule epithelium and significant alterations in germ cells. Elevated ROS levels initiate testicular germ cell and DNA damage [10,21]. Testicular tissue is particularly vulnerable to oxidative stress due to the high content of polyunsaturated fatty acids in the plasma membranes of germ cells, which are essential for maintaining sperm motility [22]. The pathogenesis of T/D-induced I/R injury involves multiple pathological mechanisms and mediators, including inflammatory cytokines and endothelial dysfunction [8]. This multifactorial nature underscores the need for therapeutic strategies targeting not only oxidative stress but also inflammation and microvascular impairment. Consequently, various antioxidants, plant extracts, chemical agents, and enzyme combinations have been investigated for their potential protective effects against I/R injury [7,9,10].

In addition to TRPM7, several members of the transient receptor potential (TRP) channel family have been implicated in oxidative stress sensing and cellular injury responses. Channels such as TRPM8, TRPV1, and TRPA1 are known to be activated directly or indirectly by ROS and play key roles in regulating intracellular calcium dynamics, inflammatory signaling, and cell fate decisions. These channels contribute to processes including mitochondrial dysfunction, apoptosis, and cellular proliferation, highlighting their broader role as integrators of stress signaling pathways. In urogenital tissues, particularly in the prostate, TRP channels have also been associated with androgen signaling, calcium homeostasis, and tumor cell survival, further supporting their relevance in tissue-specific pathophysiology. Therefore, the observed TRPM7 upregulation in the present study may reflect a component of a more extensive TRP-mediated stress response network.

Consistent with the literature, our histopathological evaluation revealed increased interstitial edema, degeneration of the germinal epithelium, atypical residual bodies, sloughing of germ cells, and the presence of multinucleated giant cells in the T/D groups. Similar findings, including necrotic changes in spermatogenic cells, structural degeneration of seminiferous tubules, congestion, and disruption of the blood–testis barrier, have been reported in previous experimental torsion studies [7,10]. These structural alterations are indicative of impaired spermatogenesis and may have long-term implications for male fertility.

The principal components of TOS include hydrogen peroxide and lipid hydroperoxides. Lipid peroxidation and its byproducts are known to damage cellular membranes and increase membrane permeability, leading to dysfunction of various cellular components [21,23]. Testicular torsion has been reported to induce hypoxic conditions that enhance oxidative stress and facilitate the accumulation of toxic metabolites, ultimately leading to germ cell death. In line with these findings, our results demonstrated increased TAS and decreased TOS levels in the T/D + iloprost group compared with the untreated T/D group, suggesting that the beneficial effects of iloprost may be attributed to its antioxidant and vasodilatory properties [24,25]. These findings support the hypothesis that iloprost mitigates systemic oxidative stress, although this biochemical improvement does not necessarily translate into structural or cellular recovery within the examined timeframe.

Exposure to oxidant agents has been shown to upregulate TRPM7 expression, and TRPM7 has been implicated in I/R injury in organs such as the brain and kidney. Meng et al. demonstrated that TRPM7 protein expression significantly increased in the kidneys of rats subjected to I/R injury during the early period (1–24 h), followed by a decline at 48 h and normalization at 72–96 h compared with controls [26]. More importantly from a mechanistic perspective, Liu et al. reported increased TRPM7 expression and TRPM7-like currents during renal I/R-related injury and demonstrated that TRPM7 silencing attenuated caspase-3 activation, the BAX/BCL-2 ratio, apoptosis, and tissue injury [17]. Thus, previous studies involving direct TRPM7 manipulation provide evidence that TRPM7 can functionally contribute to apoptotic injury in certain I/R models.

In our testicular T/D model, increased TRPM7 expression occurred concurrently with increased TOS, BAX expression, the BAX/BCL-2 ratio, and apoptotic cell ratio. This parallel pattern is consistent with, but does not prove, a possible involvement of TRPM7 in testicular I/R-associated apoptosis. Because TRPM7 was not inhibited or silenced in the present study, the direction and causality of this relationship cannot be determined from our data.

Importantly, the RT-qPCR findings further supported an association between increased TRPM7 expression and apoptosis in testicular torsion–detorsion injury. TRPM7 mRNA expression was significantly increased in the T/D groups, paralleling the marked upregulation of the pro-apoptotic marker BAX. The concurrent elevation of TRPM7 expression, apoptotic cell ratio (ACR), BAX expression, and the BAX/BCL-2 ratio demonstrates that increased TRPM7 expression and apoptotic changes occur concurrently during testicular I/R injury. However, this parallel response should not be interpreted as evidence that TRPM7 directly induces germ cell apoptosis. Because TRPM7 activity and intracellular calcium dynamics were not directly assessed and TRPM7 was not experimentally inhibited or silenced, the present findings do not establish whether TRPM7 functionally contributes to germ cell apoptosis. In contrast, the anti-apoptotic marker BCL-2 showed no substantial compensatory increase, further supporting the predominance of pro-apoptotic mechanisms in the injured testicular tissue. Although iloprost improved systemic oxidative stress parameters, it did not significantly alter TRPM7 or BAX expression, suggesting a dissociation between the improvement in systemic oxidative status and the persistence of local molecular and apoptotic alterations within the examined time frame.

The BAX/BCL-2 ratio is widely regarded as an important indicator of the balance between pro-apoptotic and anti-apoptotic signaling, with an increased ratio reflecting a shift toward apoptosis [27]. In the present study, the increased BAX/BCL-2 ratio in the T/D group supports the activation of pro-apoptotic mechanisms following testicular ischemia–reperfusion injury. Notably, iloprost treatment did not significantly reduce the BAX/BCL-2 ratio compared with the T/D group. Together with the persistence of an increased ACR, this finding indicates that the improvement in systemic oxidative status produced by iloprost was not accompanied by a significant attenuation of the apoptotic parameters evaluated in the present study. However, because downstream apoptotic mediators were not assessed, these findings do not exclude possible effects of iloprost on other components of apoptotic signaling.

Iloprost, a synthetic analog of prostacyclin (PGI_2_), is known for its antioxidant, vasodilatory, and antiaggregatory properties [28]. It also exerts antithrombotic and anti-inflammatory effects, including inhibition of platelet activation and modulation of pro-inflammatory cytokine production [29,30]. Importantly, iloprost was not selected in the present study as a TRPM7 inhibitor, and our experimental design did not assume a direct pharmacological interaction between iloprost and TRPM7. Rather, iloprost was selected because its vasodilatory, microcirculatory, anti-inflammatory, and antioxidant-related properties are relevant to the pathophysiology of I/R injury. In addition, Canacankatan et al. demonstrated that iloprost attenuated caspase-3, caspase-8, and caspase-9 activities in an experimental renal I/R model, providing evidence that its protective actions may also be associated with modulation of apoptotic signaling [20].

Accordingly, our aim was to determine whether improvement of the I/R environment by iloprost would be accompanied by changes in TRPM7 expression and apoptosis, rather than to test iloprost as a direct TRPM7-targeting agent. In our study, iloprost administration resulted in decreased serum TOS levels and increased TAS levels, supporting its antioxidant effects. However, despite these biochemical improvements, iloprost did not significantly reduce TRPM7 immunoreactivity, ACR, the BAX/BCL-2 ratio, or histopathological injury compared with the untreated T/D group. Thus, under the present experimental conditions, improvement in systemic oxidative status was not accompanied by significant suppression of TRPM7-associated expression changes or the evaluated apoptotic endpoints. This finding should not be interpreted as evidence for or against a direct effect of iloprost on TRPM7 channel function, because TRPM7 activity was not measured.

Several factors may explain the limited histological and anti-apoptotic effects of iloprost observed in this study. First, the evaluation was performed 48 h after a single-dose administration, which may have been insufficient to achieve sustained tissue protection. Second, the selected dose and route of administration might not have provided adequate local drug concentrations within the testicular tissue. Third, apoptotic and structural recovery processes often require longer time periods, suggesting that extended treatment protocols or repeated dosing may yield different outcomes. These considerations indicate the need for further experimental studies to optimize dosing strategies and therapeutic timing.

Another consideration is the timing of iloprost administration relative to detorsion. In the present study, iloprost was administered immediately before detorsion, when testicular blood flow was still markedly compromised by torsion. Therefore, drug delivery and local accumulation within the severely ischemic testicular tissue may have been limited before restoration of blood flow. Although reperfusion following detorsion would subsequently facilitate drug delivery to the tissue, early reperfusion-associated cellular events may occur rapidly. Thus, the possibility that the timing of administration influenced the local protective efficacy of iloprost cannot be excluded. Since tissue concentrations of iloprost were not measured in the present study, this interpretation remains speculative. Future studies comparing different administration schedules, including pretreatment before torsion and repeated dosing after detorsion, may help determine whether the timing of iloprost administration influences its protective effects on testicular I/R injury [31].

This study has several limitations that should be acknowledged. The principal limitation of this study is the absence of direct functional manipulation of TRPM7. TRPM7 was evaluated at the mRNA and immunohistochemical expression levels, whereas channel activity was not measured and TRPM7 was not pharmacologically inhibited or genetically silenced. Consequently, the present study cannot establish whether increased TRPM7 expression is a cause, a consequence, or an accompanying feature of apoptotic injury following testicular torsion–detorsion. Although previous studies in other ischemic/hypoxic models have demonstrated that TRPM7 suppression or silencing can reduce Ca^2+^ influx, ROS generation, caspase activation, the BAX/BCL-2 ratio, and apoptosis [15,32], equivalent functional evidence is not yet available from the present testicular model. Direct TRPM7 inhibition or genetic silencing will therefore be essential to determine whether TRPM7 is mechanistically involved in germ cell apoptosis following testicular I/R injury.

Second, although apoptotic changes were evaluated by TUNEL assay and BAX/BCL-2 expression, additional markers of apoptotic signaling, including cleaved caspase-3, PARP cleavage, cytochrome c release, and mitochondrial membrane potential, were not evaluated. Therefore, the present findings do not allow for detailed characterization of the downstream apoptotic pathways potentially involved in testicular I/R injury. Future studies incorporating these markers are required to provide a more comprehensive mechanistic assessment of apoptosis.

Third, oxidative stress assessment was limited to TAS and TOS measurements, and the inclusion of specific antioxidant enzymes such as superoxide dismutase, catalase, and glutathione peroxidase, as well as mitochondrial ROS markers, would provide a more comprehensive evaluation.

Fourth, the analysis was performed at a single time point (48 h), whereas I/R injury is a dynamic process and TRPM7 expression may vary temporally. Finally, tissue concentrations of iloprost were not measured, and the present study cannot determine whether the timing of administration or limited delivery to the ischemic testis influenced its local effects.

Future studies incorporating direct functional assessment and modulation of TRPM7, time-course analyses, measurements of intracellular Ca^2+^/Mg^2+^ dynamics and mitochondrial function, and more detailed apoptotic signaling analyses are warranted to clarify the mechanistic role of TRPM7 in testicular I/R injury.

## 4. Materials and Methods

**Ethical Approval**

The study was initiated following approval from the Firat University Local Ethics Committee for Animal Experiments (Approval No: 2018/05-68). All experimental procedures were conducted in accordance with institutional and international guidelines for the care and use of laboratory animals. The experiments were carried out in the Histology and Embryology Research Laboratory of Firat University.

**Experimental Animals and Housing Conditions**

A total of 35 male Sprague Dawley rats, aged 8–10 weeks, were obtained from the Firat University Experimental Research Center. The animals were housed in specially designed cages under controlled environmental conditions with a 12 h light/12 h dark cycle (lights on at 07:00 and off at 19:00) and an ambient temperature of 22–25 °C. Rats were provided with standard laboratory chow and tap water **ad libitum**. Feed was supplied in stainless steel containers and water in glass bottles. The cages were cleaned daily.

**Sample Size Calculation**

Sample size estimation was performed using G*Power software (version 3.1.9.7; Heinrich Heine University, Düsseldorf, Germany). An a priori power analysis for a one-way ANOVA with five independent groups, assuming a large effect size (f = 0.40), a significance level of α = 0.05, and a statistical power of 80% (1–β = 0.80), indicated that a minimum total sample size of 35 animals was required. Accordingly, seven rats were included in each group.

**Randomization and Animal Exclusion**

Prior to the experimental procedures, the animals were randomly allocated to five experimental groups (*n* = 7 per group) using simple random allocation. No animals died during the experimental period, and no animals were excluded from the study or subsequent analyses. Therefore, all 35 animals initially included in the study were included in the final analyses.

**Experimental Design and Grouping**

The rats were randomly assigned to five groups, each consisting of seven animals (*n* = 7):

**Group I (Control):** No surgical or pharmacological intervention was performed throughout the experimental period.

**Group II (Sham):** Following laparotomy, the left testis was exteriorized and maintained outside the abdominal cavity for 60 min without inducing torsion. The testis was then repositioned into the abdominal cavity, and the incision was closed.

**Group III (Iloprost):** Rats received a single intraperitoneal (i.p.) dose of iloprost (2 µg/kg) without any surgical intervention. The iloprost dose of 2 µg/kg was selected based on previous experimental studies in rats in which this dose was used to evaluate the tissue-protective effects of iloprost [33]. Iloprost (Ilomedin^®^; Schering, Berlin, Germany) was administered immediately before detorsion to ensure its availability at the onset of reperfusion and to evaluate its potential protective effects against the early oxidative and inflammatory events associated with reperfusion injury.

**Group IV (Torsion–Detorsion):** Following laparotomy, the left testis was exteriorized and rotated 720° counterclockwise to induce torsion. Torsion was maintained for 60 min, after which detorsion was performed, and the testis was returned to the abdominal cavity before closure. The testicular torsion–detorsion procedure was selected to reproduce the ischemia–reperfusion injury that occurs clinically following restoration of blood flow to a torsed testis. A 720° torsion was used to induce marked testicular ischemia, followed by detorsion to initiate reperfusion and the associated I/R injury. This experimental approach has been widely used in rat models to investigate the pathophysiological consequences of testicular torsion–detorsion and potential protective interventions [33,34]. In the present study, the 60-min torsion period was therefore employed to induce testicular ischemia while allowing subsequent evaluation of reperfusion-associated tissue injury.

**Group V (Torsion–Detorsion + Iloprost):** The torsion procedure described for Group IV was applied. At the end of the 60 min torsion period, a single intraperitoneal dose of iloprost (2 µg/kg) was administered immediately before detorsion, and the testis was then repositioned into the abdominal cavity.

**Figure 8:**
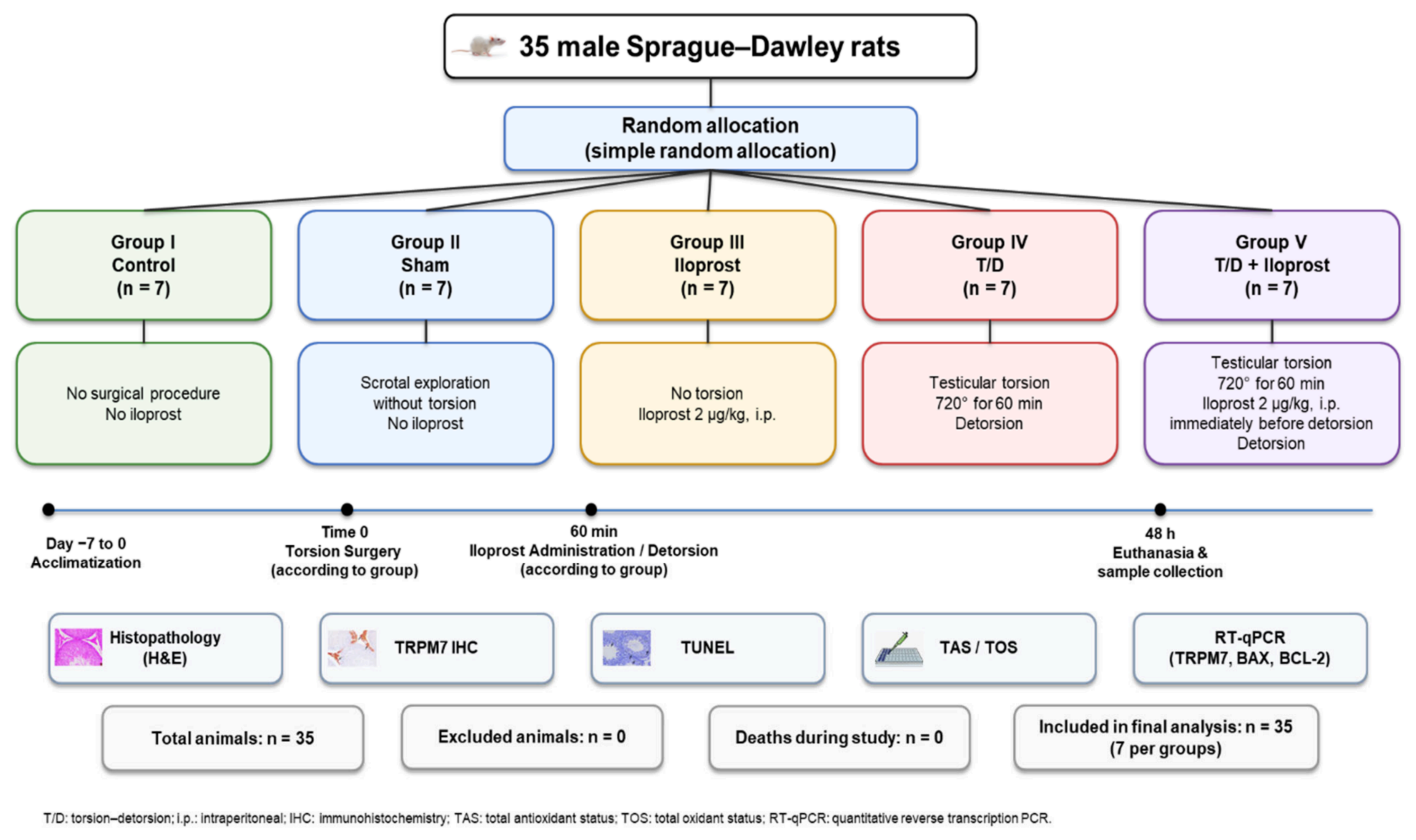
**Schematic timeline and flowchart of the experimental design.** A total of 35 male Sprague Dawley rats were randomly allocated to five experimental groups (*n* = 7 per group): Control, Sham, Iloprost, T/D, and T/D + Iloprost. Testicular torsion was induced by 720° rotation for 60 min, followed by detorsion. In the T/D + Iloprost group, iloprost (2 µg/kg, i.p.) was administered immediately before detorsion. Animals were euthanized 48 h after the experimental procedures for sample collection and subsequent analyses. No animals died or were excluded during the study (final *n* = 35). T/D, torsion–detorsion; IHC, immunohistochemistry; RT-qPCR, quantitative reverse transcription polymerase chain reaction; TAS, total antioxidant status; TOS, total oxidant status.

All experimental procedures were conducted during the light phase, between 09:00 and 12:00. A schematic timeline of the experimental design, including group allocation, experimental interventions, the 48 h observation period, sample collection, and the number of animals included and excluded from the study, is presented in Figure 8.

**Anesthesia and Euthanasia**

At the end of the 48-h experimental period, all animals were anesthetized with intraperitoneal ketamine (75 mg/kg) and xylazine (10 mg/kg) and subsequently euthanized by decapitation under deep anesthesia.

**Tissue and Blood Sample Collection**

Following euthanasia, testicular tissues were excised for histological and immunohistochemical analyses and immediately fixed in Bouin’s solution. Blood samples were collected into gel-containing biochemical tubes, centrifuged at 3500 rpm for 5 min, and the sera were separated and stored at −80 °C until biochemical analyses.

**Histological Examination**

After fixation in Bouin’s solution, tissue samples were washed in a graded alcohol series and processed using routine histological procedures. Following routine tissue processing and paraffin embedding, sections of 4–6 µm thickness were obtained from the paraffin blocks and used for hematoxylin and eosin (H&E) staining and immunohistochemical (IHC) analyses and examined under a light microscope (Leica DM500, Leica Biosystems, Nussloch, Germany). Representative images were captured using a Leica DFC295 digital camera. Histopathological alterations were evaluated semi-quantitatively using a 0–3 scoring system according to the severity of tissue damage: 0, no histopathological alteration; 1, mild alteration; 2, moderate alteration; and 3, severe alteration. The scoring was performed based on the overall degree of histopathological changes observed in the testicular tissue.

**TUNEL Assay for Detection of Apoptosis**

Apoptotic cells were detected using the **ApopTag Plus Peroxidase In Situ Apoptosis Detection Kit** (Cat. No: S7101, Chemicon, Merck KGaA, Burlington, MA, USA) according to the manufacturer’s instructions. Paraffin sections (4–6 µm) mounted on poly-L-lysine-coated slides were deparaffinized in xylene, rehydrated through graded alcohols, and washed with phosphate-buffered saline (PBS). Sections were incubated with 0.05% proteinase K for 10 min, followed by 3% hydrogen peroxide for 5 min to block endogenous peroxidase activity. After incubation with equilibration buffer for 6 min, sections were treated with the working solution (70% reaction buffer + 30% TdT enzyme) at 37 °C for 60 min in a humidified chamber. The reaction was terminated using stop/wash buffer for 10 min. Subsequently, sections were incubated with anti-digoxigenin–peroxidase for 30 min and visualized using diaminobenzidine (DAB). Counterstaining was performed with Harris hematoxylin.

Cells exhibiting brown nuclear staining were considered apoptotic, whereas blue-stained nuclei were regarded as normal. For the negative control, the TdT enzyme was omitted and replaced with reaction buffer. In randomly selected fields at ×10 magnification, at least 500 cells were counted, and the **Apoptotic Index (AI)** was calculated as follows:

$$AI\left(\%\right)=\frac{\mathrm{Number}\;\mathrm{of}\;\mathrm{apoptotic}\;\mathrm{cells}}{\mathrm{Total}\;\mathrm{number}\;\mathrm{of}\;\mathrm{cells}\;(\mathrm{normal}\;+\;\mathrm{apoptotic})}\times 100$$

**Immunohistochemical Analysis of TRPM7**

Paraffin sections (4–6 µm) mounted on poly-L-lysine-coated slides were deparaffinized and rehydrated. Antigen retrieval was performed in citrate buffer (pH 6.0) using a microwave oven at 750 W for 15 min, followed by cooling at room temperature for 25 min. After washing with PBS (P4417, Sigma-Aldrich, St. Louis, MO, USA), endogenous peroxidase activity was blocked using Hydrogen Peroxide Block (TA-125-HP, Lab Vision Corporation, Fremont, CA, USA) for 5 min. Non-specific binding was prevented by incubation with Ultra V Block (TA-125-UB, Lab Vision Corporation, Fremont, CA, USA) for 5 min.

Sections were incubated with **goat anti-TRPM7 primary antibody** (ab729, Abcam, Cambridge, UK) diluted 1:200 for 60 min at room temperature in a humidified chamber. After PBS washing, sections were treated with **donkey anti-goat secondary antibody** (sc-2042, Santa Cruz Biotechnology, Dallas, TX, USA) for 30 min, followed by **streptavidin–peroxidase** (TS-125-HR, Lab Vision Corporation, Fremont, CA, USA) for another 30 min. Immunoreactivity was visualized using **3-amino-9-ethylcarbazole (AEC)** substrate and chromogen. Sections were counterstained with Mayer’s hematoxylin and mounted using Large Volume Vision Mount (TA-125-UG, Lab Vision Corporation, Fremont, CA, USA). Slides were examined and photographed using a Leica DM500 microscope equipped with a Leica DFC295 digital camera.

Immunoreactivity was evaluated semi-quantitatively using a histoscore method calculated as the product of distribution and staining intensity. Distribution was graded as 0.1 for <25%, 0.4 for 26–50%, 0.6 for 51–75%, and 0.9 for 76–100% of positive cells. Staining intensity was scored as 0 (none), 0.5 (very weak), 1 (weak), 2 (moderate), and 3 (strong). The final histoscore was obtained by multiplying the distribution score by the intensity score [35].

A separate negative control, such as omission of the primary antibody, was not included in the immunohistochemical staining procedure.

**Biochemical Analyses**

Serum total antioxidant status (TAS) and total oxidant status (TOS) were measured using enzyme-linked immunosorbent assay (ELISA) kits (YL Biotechnology Co., Ltd., Shanghai, China) according to the manufacturer’s instructions. The TAS kit (Cat. No. YLA3389Ra, YL Biont, Shanghai, China) had a measurement range of 1–300 pg/mL, with a sensitivity of 0.54 pg/mL, an intra-assay coefficient of variation (CV) <10%, and an inter-assay CV <12%. The TOS kit (Cat. No. YLA1392Ra, YL Biont, Shanghai, China) had a measurement range of 0.02–60 U/mL, with a sensitivity of 0.013 U/mL, an intra-assay CV <10%, and an inter-assay CV <12%. Plate washing was performed using an automated microplate washer (BioTek ELx50, BioTek Instruments, Winooski, VT, USA), and absorbance was measured using a microplate reader (ChroMate P4300, Awareness Technology, Palm City, FL, USA). All results are expressed as U/mL.

**Quantitative Real-Time PCR (qPCR)**

Total RNA was isolated from rat testicular tissues using Tri Reagent (Cat. No. NGE023, Nucleogene, Istanbul, Türkiye) according to the manufacturer’s instructions. Tissue samples were homogenized in Tri Reagent using stainless steel beads (SSB32, Next Advance, Troy, NY, USA) in a tissue homogenizer (Bullet Blender Storm 24, Next Advance, Troy, NY, USA) to ensure efficient cell lysis. RNA purity and concentration were assessed using a microspectrophotometer (AMR100, Allsheng Instruments, Hangzhou, China), and only RNA samples with A260/A280 ratios between 1.8 and 2.2 were included in the analysis. RNA integrity was further verified by agarose gel electrophoresis. To eliminate potential genomic DNA contamination, samples were treated with DNase I (Cat. No. M0303S, New England Biolabs, Ipswich, MA, USA). First-strand complementary DNA (cDNA) was synthesized from 1 µg of total RNA using a cDNA synthesis kit (Vitascript, Procomcure Biotech GmbH, Thalgau, Austria), following the manufacturer’s protocol. The reaction was performed in a total volume of 20 µL using random hexamer and oligo(dT) primers in a thermal cycler (Veriti, Applied Biosystems, Foster City, CA, USA). Quantitative real-time PCR was carried out using SYBR Green Master Mix (Cat. No. NGMM007, Nucleogene, Istanbul, Türkiye) on an ABI 7500 Real-Time PCR system (Applied Biosystems, Singapore). Each reaction was performed in a final volume of 20 µL containing 10 µL of SYBR Green Master Mix, 2 µL of cDNA template, and 0.5 µM of each forward and reverse primer. All reactions were conducted in triplicate. The thermal cycling conditions were as follows: an initial denaturation step at 95 °C for 10 min, followed by 40 cycles of denaturation at 95 °C for 15 s and annealing/extension at 60 °C for 40 s. A melt curve analysis was performed at the end of each run to confirm the specificity of amplification. Gene-specific primer sequences are listed in Table 1. Amplification efficiency for each primer pair was validated and found to be within the acceptable range (90–110%). No-template controls (NTCs) and no-reverse transcription (no-RT) controls were included to exclude contamination and genomic DNA amplification. Beta-actin (ACTB) was used as the reference gene after confirming its stable expression across experimental groups. Relative gene expression levels were calculated using the 2^−ΔΔCt^ method, with the control group serving as the calibrator.

## 5. Blinding

Due to the nature of the experimental interventions, the investigator performing the torsion–detorsion procedure and iloprost administration was aware of the group assignments. To minimize assessment bias, blinding was applied during outcome evaluation. Tissue sections were coded with numerical identifiers, and the investigator performing the histopathological assessment was unaware of the corresponding experimental groups. TRPM7 immunohistochemical and TUNEL evaluations were also performed under blinded conditions using coded sections. Similarly, the investigators performing the ELISA and RT-qPCR analyses were unaware of the group assignments during the analyses. **The statistical analysis was not performed under blinded conditions.** Group identities for the blinded assessments were disclosed only after completion of the respective evaluations.

## 6. Statistical Analysis

Statistical analyses were performed using GraphPad Prism version 8.4.2 (GraphPad Software, San Diego, CA, USA). Data distribution was assessed using the Shapiro–Wilk normality test. Homogeneity of variances was evaluated where appropriate using the Brown–Forsythe test. The choice of statistical test was based on the distributional characteristics and measurement scale of the data.

For normally distributed continuous data satisfying the assumptions of parametric analysis, differences among the five experimental groups were evaluated using ordinary one-way analysis of variance (one-way ANOVA), followed by Tukey’s multiple-comparison test. Data analyzed using parametric methods are presented as mean ± standard deviation (SD), together with individual data points in the corresponding graphs. For data that did not satisfy the assumptions of parametric analysis, as well as ordinal/semi-quantitative histopathological and immunohistochemical scores, differences among groups were assessed using the Kruskal–Wallis test followed by Dunn’s multiple-comparison test. These data are presented as medians (min–max) where appropriate, and individual observations are displayed in the corresponding graphs.

For qRT-PCR analyses, relative mRNA expression was calculated using the 2^−ΔΔCt^ method, with Group I serving as the calibrator. Statistical comparisons of TRPM7, BAX, and BCL-2 expression were performed on the corresponding ΔCt values rather than on the fold-change values. After assessment of normality and homogeneity of variances, ΔCt values were analyzed using ordinary one-way ANOVA followed by Tukey’s multiple-comparison test. The 2^−ΔΔCt^ values were used for graphical presentation of relative mRNA expression, with individual animals shown as separate data points and summary values presented as mean ± SD.

The relative BAX/BCL-2 expression ratio was calculated individually for each animal from the corresponding BAX and BCL-2 relative expression values. Because the log_2_-transformed BAX/BCL-2 ratio values did not satisfy the assumptions of parametric analysis, differences among groups were evaluated using the Kruskal–Wallis test followed by Dunn’s multiple-comparison test. Individual BAX/BCL-2 ratio values are graphically presented with the median.

No formal outlier exclusion test was applied, and no data points were excluded from the analyses. All measurements obtained from the seven animals in each group were retained and included in the statistical analyses. Statistical significance was set at *p* < 0.05. For graphical presentation of multiple comparisons, different lowercase letters indicate statistically significant differences between groups, whereas groups sharing at least one letter are not significantly different.

## 7. Conclusions

In conclusion, testicular torsion–detorsion decreased TAS levels while increasing TOS levels, TRPM7 expression, apoptosis, and histopathological alterations. Iloprost administration restored TAS and TOS levels toward control values; however, it did not exert notable effects on TRPM7 expression, apoptosis, or histopathological damage under the experimental conditions used. These findings indicate that increased TRPM7 expression is associated with testicular ischemia–reperfusion injury; however, the present study does not establish a causal relationship between TRPM7 and apoptotic cell death. Because TRPM7 channel activity was not directly assessed and TRPM7 was not pharmacologically inhibited or genetically silenced, it cannot be determined whether increased TRPM7 expression is a cause, consequence, or accompanying feature of apoptotic injury. Furthermore, iloprost was not used as a TRPM7 inhibitor, and the present findings do not support a direct mechanistic interaction between iloprost and TRPM7. Further functional studies involving direct modulation of TRPM7 are required to determine whether TRPM7 plays a causal role in apoptosis. In addition, studies investigating different iloprost doses, administration schedules, and longer observation periods are warranted to better elucidate its therapeutic potential in testicular ischemia–reperfusion injury.

## Figures and Tables

**Table 1:** Gene-specific primer sequences used for quantitative real-time PCR.

Symbol	Primer Sequence (5′–3′)		Size (bp)	Accession No
ACTB	F	ACTCTGTGTGGATTGGTGGC	140	NM_031144.3
	R	CGCAGCTCAGTAACAGTCCG		
TRPM7	F	TGGGTAAAGGTGGAAAAGAAAGG	187	XM_039105837.2
	R	AGACATCTGTGAGGGTCTTTGG		
BAX	F	CATGTCTCTGTTCTCAGGATGGC	198	NM_017059.2
	R	TCCCCGTTCCCCATTCATCC		
BCL-2	F	ATGACTTCTCTCGTCGCTACC	160	NM_016993.2
	R	CCACCGAACTCAAAGAAGGC		

Abbreviations: F—forward primer; R—reverse primer.

## Data Availability

The original contributions presented in this study are included in the article. Further inquiries can be directed to the corresponding author.
